# Relationships between 25-Hydroxyvitamin D Levels and Obstructive Sleep Apnea Severity in Children: An Observational Study

**DOI:** 10.3390/jcm12031242

**Published:** 2023-02-03

**Authors:** Cristian Locci, Antonella Ruiu, Laura Saderi, Giovanni Sotgiu, Stefania Bassu, Marco Zaffanello, Roberto Antonucci

**Affiliations:** 1 Pediatric Clinic, Department of Medicine, Surgery and Pharmacy, University of Sassari, 07100 Sassari, Italy; 2 Clinical Epidemiology and Medical Statistics Unit, Department of Medicine, Surgery and Pharmacy, University of Sassari, 07100 Sassari, Italy; 3 Department of Biomedical Sciences, University of Sassari, Viale San Pietro 43, 07100 Sassari, Italy; 4 Department of Surgical Sciences, Dentistry, Gynecology and Pediatrics, University of Verona, 37129 Verona, Italy

**Keywords:** vitamin D, hypovitaminosis D, obstructive sleep apnea syndrome, children

## Abstract

The prevalence of hypovitaminosis D is increasing worldwide. Vitamin D deficiency is supposed to play a role in sleep disturbances, but the complex relationships between hypovitaminosis D and pediatric obstructive sleep apnea syndrome (OSAS) are still incompletely understood. This study was aimed to retrospectively investigate the vitamin D status and significant clinical, laboratory, and instrumental variables in a cohort of pediatric patients with OSAS and to assess the possible relationship between serum vitamin D levels and OSAS severity. We consecutively enrolled all children aged 2–14 years admitted to our Pediatric Clinic from 1 July 2018 to 30 November 2020 for sleep-disordered breathing. Each patient underwent standard overnight in-hospital polygraphic evaluation, measurement of serum 25-hydroxyvitamin D (25(OH)D) levels, and clinical and laboratory investigation. A total of 127 children with OSAS were included. The 25(OH)D levels and BMI of OSAS patients were compared with those of an age-matched control group: the serum 25(OH)D levels were significantly lower in OSAS patients than in controls (22.4 vs. 25.5 ng/mL; *p*-value = 0.009), whereas no differences in the BMI percentile were found between the two groups. The mean value of 25(OH)D was not significantly lower (20.9 ng/mL) in the severe OSAS group compared with the mild (23.0 ng/mL) and moderate (23.3 ng/mL) OSAS groups (*p*-value = 0.28). Our findings indicate a relationship between vitamin D status and OSAS in children and suggest that severe cases of OSAS have lower vitamin D levels. Future, more extensive prospective studies are needed to confirm such preliminary findings.

## 1. Introduction

Hypovitaminosis D appears to have an increasing prevalence worldwide. Low vitamin D levels were found to be associated with the hospital length of stay in children with non-critical illnesses [1], suggesting that the vitamin D levels could predict disease severity. Conversely, increased serum levels of vitamin D were associated with a lower risk of hospitalization for bronchiolitis [2].

Vitamin D is reported to have anti-inflammatory properties, and its deficiency is supposed to be involved in respiratory sleep disturbances [3]. It has been proposed that vitamin D deficiency may have a role in increasing the risk of obstructive sleep apnea syndrome (OSAS) by promoting adenotonsillar hypertrophy, reduced airway muscle tone, and/or chronic rhinitis [4,5]. OSAS is characterized by intermittent upper airway obstruction during sleep, which can lead to intermittent hypoxia, hypercapnia, increased respiratory effort with marked intrathoracic pressure swings, and repeated arousals causing sleep fragmentation [6]. OSAS should be considered in the same way as inflammatory diseases because intermittent hypoxia and apneic events are associated with the overexpression of inflammatory markers and increased sympathetic system activation [7]. OSAS shares important risk factors and comorbidities with vitamin D deficiency; moreover, inflammation plays a significant pathogenic role in both conditions [8]. Vitamin D suppresses the production of pro-inflammatory cytokines including IL-2, INFγ, and TNFα and promotes the secretion of anti-inflammatory cytokines such as IL-3, IL-4, IL-5, and IL-10. From this, it can be inferred that its deficiency is related to an increased propensity for autoimmunity and infection susceptibility [9]. Indeed, vitamin D potentiates the antimicrobial activity of macrophages and monocytes, which is crucial in the response to infections [10,11]. Low serum levels of 25(OH)D have been found to be associated with a higher incidence of upper respiratory tract infections, chronic obstructive pulmonary disease (COPD), rhinitis, and allergic asthma [10]. Recently, it has been reported that hypovitaminosis D and OSAS can worsen the asthma control in obesity-related asthmatic children [12]. Immune dysregulation and recurrent infections may contribute to developing adenotonsillar hypertrophy and chronic rhinitis, which are relevant risk factors for OSAS [13,14]. On the other hand, OSAS can represent a risk factor for hypovitaminosis D. In fact, children with OSAS often have excessive daytime sleepiness, obesity, limited access to outdoor activities, and less sunlight exposure, the latter being an essential factor for the endogenous synthesis of this vitamin [15].

Ozgurhan et al. [16] performed a prospective study to assess the risk of OSAS in subjects with vitamin D deficiency. The authors recruited 240 subjects aged 7–14 years, who were divided into two groups: one group (*n* = 120) with 25(OH)D levels <20 ng/mL, and the second one (*n* = 120) with 25(OH)D levels >20 ng/mL (control group). The risk of developing OSAS was significantly higher in the group with a low level (<20 ng/mL) of 25(OH)D compared to the control group (*p*-value = 0.030). Moreover, in patients with OSAS, the serum vitamin D levels are inversely proportional to hypoxia indices such as the apnea–hypopnea index (AHI), the oxygen desaturation index (ODI), and the total sleep time with oxygen saturation < 90% (TST-SpO_2_ < 90%) [17].

It has been proposed that in OSAS patients, the inflammatory process influences the regulation of thrombopoiesis, inducing an increase in MPV and PLT as the inflammation develops [18]. An Italian study provided evidence of higher mean platelet volume (MPV), higher platelet counts (PLT), and lower vitamin D levels in children with OSAS, supporting the existence of an underlying inflammation [19].

Epidemiological studies showed that pediatric obesity represents an important risk factor for OSAS, especially among adolescents [12]. In the observational cross-sectional study by Kheirandish-Gozal et al. [20], 176 prospectively enrolled children (mean age, 6.8 ± 0.8 years) were subjected to overnight polysomnography. The plasma 25(OH)D concentrations were significantly lower in African American children, OSAS children, and obese children. Moreover, significant linear correlations were observed between 25(OH)D concentrations and body mass index (BMI) z-score, high sensitivity C-reactive protein (hs-CRP), homeostatic model of insulin resistance (HOMA-IR), AHI, and SpO_2_ nadir. More recently, Bhatt et al. [21] enrolled 247 children, 190 of whom with OSAS and 57 healthy controls, with a mean age of 10.71 ± 3.00 years and 11.87 ± 2.66 years, respectively. The mean values of weight and BMI were significantly higher in the OSAS patients than in the controls. Moreover, the levels of inflammatory markers including IL-6, IL-8, IL-17, IL-18, Hs-CRP, and TNFα were increased in the subjects with OSAS and significantly correlated with the AHI values.

Overall, the complex relationships between hypovitaminosis D and pediatric OSAS are still incompletely understood, and the available data are limited and not conclusive.

The present retrospective study was aimed to investigate vitamin D status, significant clinical, laboratory, and instrumental variables, and the complex relationship between the serum levels of vitamin D and OSAS severity, in a cohort of pediatric patients affected by OSAS.

## 2. Materials and Methods

From 1 July 2018 to 30 November 2020, we consecutively enrolled Caucasian children aged 2–14 years referred to the pediatric pulmonology service of the Pediatric Clinic of the University of Sassari, Italy, with suspicion of OSAS. Exclusion criteria were genetic or craniofacial syndromes, neuromuscular diseases, recent infections, gastrointestinal malabsorption, and inflammatory bowel diseases.

For each enrolled child, a complete medical history and physical examination were performed by a single investigator. Demographic and clinical data including gender, age, BMI percentile, tonsillar grading (Mallampati score), palate conformation (Friedman Palate Position), oral breathing, degree of nasal obstruction, and presence of inhalant allergen sensitization were obtained. The skin prick test (SPT) and total IgE were used to detect the sensitization to allergens.

Age- and sex-specific BMI percentiles based on Italian cross-sectional growth charts [22] were obtained. According to these reference growth charts, subjects with a BMI between the 85th and the 94th percentiles were classified as overweight, while those with BMI ≥ the 95th percentile were categorized as obese [23].

Currently, overnight in-lab polysomnography is the gold standard for the diagnosis of OSAS in children. Nevertheless, home cardio-respiratory polygraphy (HRP) has proven to be a potentially valuable and reliable approach [24]. All enrolled children underwent standard overnight in-hospital polygraphy (PG) (SOMNOscreen™ Plus, SOMNOmedics GmbH, Randersacker, Germany), with assessment of the following parameters: oro–nasal airflow, snoring, thoracic and abdominal movements (inductance plethysmography), pulse oximetry, and position. The results of PG were evaluated according to the American Academy of Pediatrics and American Academy of Sleep Medicine guidelines for pediatric OSAS [25,26]. AHI was calculated as the number of apnea and hypopnea episodes per hour of sleep (events/h). For the purposes of this study, the diagnosis of OSAS was defined as AHI ≥ 2 events/h of total sleep time. The severity of OSAS was classified as follows: mild OSAS (AHI < 5/h); moderate OSAS (5/h ≤ AHI < 10/h); severe OSAS (AHI ≥ 10/h). The SpO_2_ nadir is the lowest value of oxygen saturation measured in total sleep time [25,26].

In the morning, at the end of the polygraphic study, the patients underwent blood sampling to determine their serum vitamin D levels, total IgE, and complete blood count.

The serum 25(OH)D levels were measured by the laboratory using the immune-chemiluminescence LIAISON^®^ 25 OH Vitamin D Total Assay (CLIA, DiaSorin Spa, Saluggia, Italy) following the manufacturer’s instructions [27]. The vitamin D status of the participants was classified according to the Global Consensus Recommendations on Prevention and Management of Nutritional Rickets [28] as follows: vitamin D sufficiency (serum 25(OH)D > 20 ng/mL), insufficiency (serum 25(OH)D, 12–20 ng/mL), and deficiency (serum 25(OH)D < 12 ng/mL).

The cohort of OSAS patients was compared with a group of healthy controls (HC) aged 2 to 14 years, who had undergone routine medical and laboratory examinations in a pediatric primary care setting from 1 January to 31 December 2018. To exclude the influence of age, an age-matched analysis was performed between the two groups, but exclusively for BMI and serum 25(OH)D levels.

### Statistical Analysis

All collected data were entered into an electronic database, and statistical analysis was performed. Qualitative data are expressed as absolute and relative frequencies, whereas quantitative data are presented as mean and standard deviation (SD) or median and interquartile range (IQR) as appropriate. The Chi-squared test or the Fisher’s exact test was used for the comparison of the qualitative variables. The quantitative variables were compared using the Student’s *t*-test and the Mann–Whitney test for the parametric and non-parametric distributions, respectively. The ANOVA test was used to analyze differences in 25(OH)D values between the 3 groups with different severity of OSAS. Spearman’s correlation was used to evaluate the relationships between vitamin D levels, BMI percentiles, AHI, SpO_2_, PLT, and MPV. A *p*-value < 0.05 was considered statistically significant. All statistical analyses were performed using STATA software version 17 (StataCorp LLC, College Station, TX, USA).

## 3. Results

During the study period, 127 OSAS children aged 2 to 14 years (median age, 5 years) were retrospectively enrolled, with a predominance of males (60.6%). Five (3.9%) of them were classified as obese, and eleven (8.7%) as overweight.

The variables analyzed in the OSAS patients included in the study are shown in Table 1.

The mean ± SD serum 25(OH)D concentration was found to be 22.4 ± 7.7 ng/mL.

The median (IQR) BMI and the median (IQR) BMI percentile were 15.4 (14.4–17.4) kg/m^2^ and 30.5 (12–72.5), respectively.

Regarding the polygraphic parameters, the median (IQR) AHI was 7.2 events/h (5.1–12.1 events/h), whereas the median (IQR) SpO_2_ nadir was 92% (89–93%) on room air.

More than half of the OSAS patients (61.4%) had high-grade adenotonsillar hypertrophy (grade III or IV), with predominantly oral breathing. Snoring was present in 88.2% of the enrolled patients, and nasal obstruction in 81.9% of them. Rhinitis was found in less than half of the patients (41.7%), asthma was detected in 17.3% of them, while allergen sensitization was observed in about a quarter of the cases (25.2%).

The control group initially included 182 healthy Caucasian subjects (51.7% males) aged 2 to 14 years (median age, 9 years). Nine (4.9%) of them were classified as obese, and eight (4.4%) as overweight.

**Figure 1:**
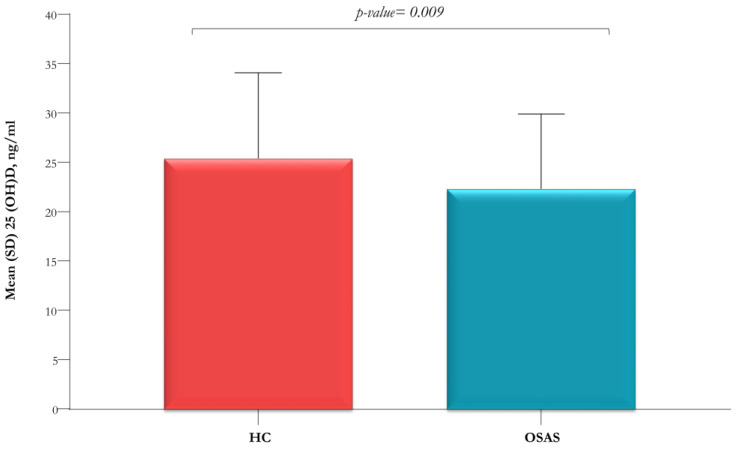
Serum vitamin D levels in OSAS patients (*n* = 96) and in age-matched healthy controls (HC) (*n* = 96).

To assess the potential role of the variable age, an age-matched analysis was performed to compare the OSAS group (*n* = 96) with HC (*n* = 96) for the only available variables BMI and serum 25(OH)D levels (Table 2). The serum level of 25(OH)D was significantly lower in children with OSAS than in HC (22.4 ± 7.6 vs. 25.5 ± 8.7 ng/mL; *p*-value = 0.009) (Figure 1). No significant differences in BMI (*p*-value = 0.19) and BMI percentile (*p*-value = 0.33) were found between the two groups.

**Figure 2:**
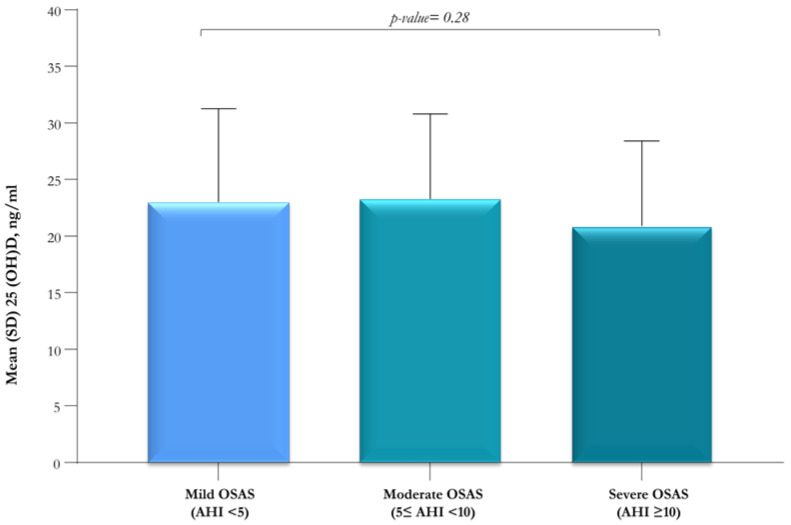
Serum 25(OH)D levels in subjects with mild (AHI < 5), moderate (5 ≤ AHI < 10), and severe (AHI ≥ 10) OSAS.

In the OSAS group, no significant differences in 25(OH)D levels were found between patients with mild (AHI < 5/h), moderate (5/h ≤ AHI < 10/h), and severe (AHI ≥ 10/h) OSAS (*p*-value = 0.28); nevertheless, the mean serum 25(OH)D level was lower (20.9 ± 7.5 ng/mL) in patients with severe OSAS when compared to those with mild (23 ng/mL ± 8.3) or moderate (23.3 ± 7.5 ng/mL) OSAS (Figure 2).

**Figure 3:**
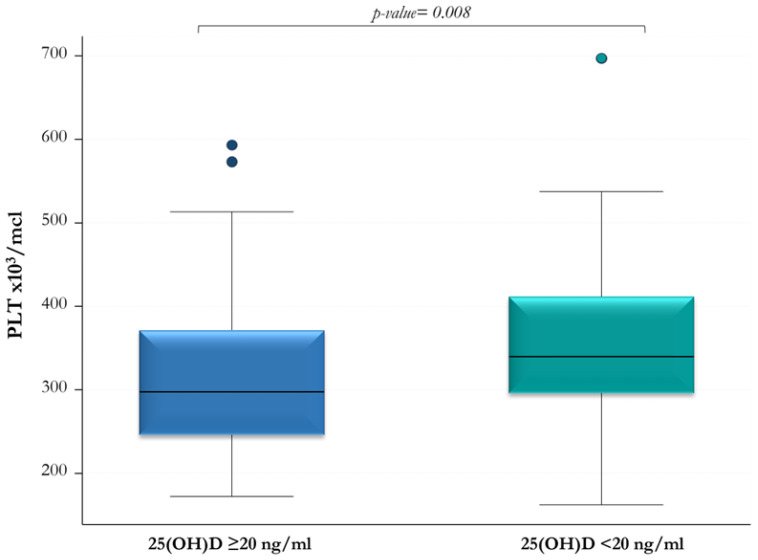
Platelet count (PLT) in OSAS patients with serum 25(OH)D levels ≥20 ng/mL and <20 ng/mL, respectively.

The OSAS patients were divided into two groups according to their vitamin D status: the first one (*n* = 44) included patients with serum 25(OH)D levels <20 ng/mL, and the second one (*n* = 83) those with 25(OH)D levels ≥20 ng/mL (Table 3). The median PLT was significantly higher in the OSAS patients with 25(OH)D levels <20 ng/mL compared to those with 25(OH)D levels ≥20 ng/mL (339.5 × 10^3^/mcl vs. 297 × 10^3^/mcl; *p*-value = 0.008) (Figure 3). No other significant differences were found between these two groups, even though a higher median AHI and a lower median SpO_2_ nadir were observed in the OSAS patients with 25(OH)D levels <20 ng/mL (Table 3).

**Figure 4:**
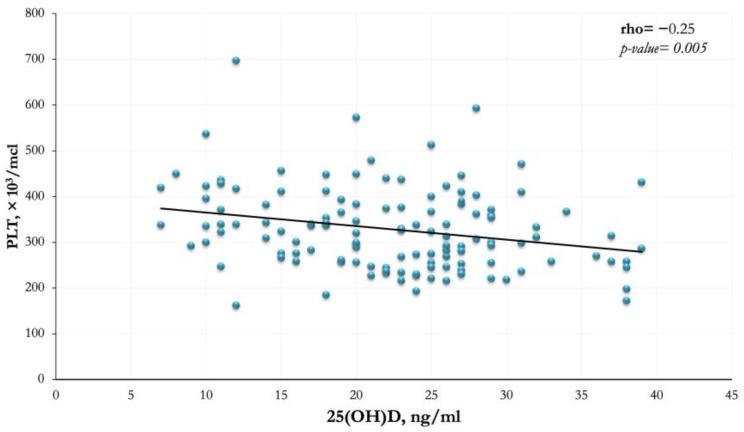
The scatterplot shows the existence of a significant negative correlation between platelet count (PLT) and serum 25(OH)D levels in the cohort of OSAS patients (rho = −0.25, *p* = 0.005). Blue points represent individual values for each participant while the black line corresponds to the linear regression.

The correlations between serum 25(OH)D levels and BMI percentile, PLT, MPV, AHI, SpO_2_ nadir in the OSAS patients were also analyzed (Table 4). A significant negative correlation (rho = −0.25; *p*-value = 0.005) between the serum levels of 25(OH)D and the median PLT was demonstrated (Figure 4).

Finally, when comparing OSAS children with allergen sensitization to those without allergen sensitization, no significant differences in AHI and serum 25(OH)D levels were found (Table 5).

## 4. Discussion

OSAS is observed throughout the entire developmental age, but it has the highest prevalence in preschool and school-aged children, coinciding with the highest frequency of adenotonsillar hypertrophy, and in adolescents, in whom obesity is more frequent. If not promptly diagnosed and treated, OSAS can lead to significant complications such as growth retardation, neurodevelopmental disorders, and, in the most severe cases, right ventricular hypertrophy and pulmonary hypertension [25]. The impact of OSAS on cognitive functions is more serious in children than in adults, as it can modify neuropsychic development, learning abilities, and social interactions by acting on a highly plastic brain [29].

In previous studies, low serum concentrations of 25(OH)D were associated with a higher incidence of upper respiratory tract infections, such as chronic rhinitis and tonsillitis, resulting in an increased tonsil and/or adenoid size. Low vitamin D levels would increase the risk of OSAS by inducing adenotonsillar hypertrophy, airway muscle myopathy, and/or chronic rhinitis [4,14,20]. In addition, a recent study showed a positive association between vitamin D deficiency and sleep architecture, suggesting a possible circadian influence, with hypovitaminosis D associated with delayed sleep onset [30].

In our study, the serum 25(OH)D levels were significantly lower in OSAS patients compared to HC matched by the variable age, consistent with literature findings [5,16,20]. More than half (61.4%) of our OSAS patients had high-grade adenotonsillar hypertrophy, with predominantly oral breathing, in line with literature data [5,31].

The possible link between OSAS and obesity has already been reported: both the set of changes accompanying obesity and those physiologically induced by sleep can explain the high cardiovascular risk, mainly when obesity and OSAS are present at the same time [32]. In the recent study by Bhatt et al. [21], children with OSAS were found to have increased obesity, insulin resistance, and systemic inflammation. Unlike previous studies on this topic, our retrospective study simultaneously investigated a significant number of clinical, laboratory, and instrumental variables in a cohort of pediatric OSAS patients with a prevalence of males (60.6%). Age- and gender-related features in pediatric OSAS have been investigated in a recent study by Kang et al. [33], which documented that male gender and obesity increase the risk of OSAS. It should be emphasized that, in the Kang’s study, the rate of obesity in the OSAS patient was much higher (21%) than that observed in our study (3.9%); moreover, we found no significant difference between OSAS patients and age-matched controls concerning the BMI percentile. Unlike what has been reported in other studies [5,16,33], the OSAS patients that we studied had a median age (IQR) of 5 years (4–7 years), an age range in which obesity is relatively infrequent. These data suggest that, in our study population, the impact of adenotonsillar hypertrophy on the development of OSAS was more prevalent than that of obesity.

Generally, obese pediatric patients with higher values of BMI show lower serum levels of 25(OH)D [5,20]. Barja-Fernández et al. [34] reported that the vitamin D levels are modulated by adiposity and that obese subjects have a reduced bioavailability of vitamin D due to its deposition in the adipose tissue. Shin et al. [5] found no significant difference in BMI between children with adenotonsillar hypertrophy and controls; moreover, the former showed a lower concentration of 25(OH)D as compared to the latter. In addition, these authors showed that the BMI z-score was negatively associated with the serum concentration of 25(OH)D. However, in this study, a definite diagnosis of OSAS based on polysomnography was not made. Kheirandish-Gozal et al. [20] observed a significant linear association between 25(OH)D plasma levels and BMI z-score in a cohort of 176 children with and without obesity or OSAS. Unlike these two studies [5,20], we found no significant correlation between 25(OH)D level and BMI percentile in OSAS patients. These conflicting results may be partly explained by the heterogeneity of the study populations regarding several variables including age range, prevalence rate of obesity, exposure to sunlight, latitude and seasonal variations.

The relationship between OSAS severity and serum 25(OH)D levels is still debated. Our study revealed that the serum 25(OH)D level in the severe OSAS group was non-significantly lower than in patients with mild or moderate OSAS. Furthermore, we observed a negative non-significant correlation between serum 25(OH)D level and AHI (rho = −0.14 *p*-value = 0.11) and a positive non-significant correlation between serum 25(OH)D level and SpO_2_ nadir (rho = 0.14, *p*-value = 0.11). These findings are in contrast to the results of Kheirandish-Gozal et al. [20], who documented significant correlations between serum level of 25(OH)D and AHI (r = −0.285, *p*-value < 0.001) and SpO_2_ nadir (r = 0.283, *p*-value < 0.001). Some studies failed to find a significant association between OSAS severity and vitamin D levels, whereas others showed that the serum levels of vitamin D were higher in non-OSAS patients and decreased with the severity of OSAS [17].

In this study, we investigated the inflammatory state in OSAS patients by using inexpensive and readily available inflammatory markers, namely, PLT and MPV. When dividing the cohort of OSAS patients into two groups according to their 25(OH)D levels, we found that PLT was significantly higher in the group with 25(OH)D levels <20 ng/mL. In contrast, the MPV values were similar in the two groups, suggesting that serum 25(OH)D did not affect this inflammatory marker. Unfortunately, the platelet parameters of HC were not available, thus not allowing their comparison between the two study groups. A significant negative correlation was found in OSAS patients between serum 25(OH)D levels and PLT, while there was no correlation between serum 25(OH)D level and MPV value. These results appear to be consistent with previous literature data. According to some studies, subjects with lower 25(OH)D concentrations and/or with more severe OSAS would have a higher PLT (reactive thrombocytosis), probably because an increased inflammatory state is not counteracted by adequate vitamin D levels [18,19]. However, studies conducted on pediatric patients provided conflicting results. Cengiz et al. [35] found low MPV values in children affected by OSAS, whereas Onder et al. [36] observed no significant correlation between MPV and OSAS. Moreover, Zicari et al. [19] documented higher values of MPV in children affected by OSAS and an inverse correlation between MPV and PLT. Other authors [37,38] recently reported higher levels of inflammatory markers (including PLT and MPV) in children with OSAS compared to healthy children.

Regarding the PG parameters, in OSAS patients with lower 25(OH)D concentrations (<20 ng/mL), we found higher AHI values, although statistical significance was not achieved. Furthermore, we observed a non-significant negative correlation between serum 25(OH)D level and AHI, whereas a significant correlation between these variables was reported by Kheirandish-Gozal et al. [20].

Literature evidence shows that low serum 25(OH)D levels are associated with atopy (rhinitis and allergic asthma) and a higher incidence of upper respiratory tract infections [39]. Both these conditions may induce the development of adenotonsillar hypertrophy, resulting in an increased risk of OSAS. In our cohort of OSAS patients, we could not demonstrate a relationship between allergen sensitization on the one hand and AHI and serum 25(OH)D level on the other.

The main limitations of this study are its retrospective observational design and the involvement of a single center. Furthermore, several variables (e.g., seasonality, dietary intake of vitamin D, outdoor activities, etc.) were not evaluated; they are key to assess the role of the confounders. Another limitation is represented by the recruitment of a control group where OSAS was not formally excluded (the diagnosis was based only on the absence of symptoms and clinical signs); this could underestimate the prevalence of OSAS in the control group.

## 5. Conclusions

The findings of the present study document a possible impact of the vitamin D status on OSAS patients, who were found to have serum 25(OH)D concentrations significantly lower compared to HC; moreover, the lowest levels of vitamin D were detected in patients with severe OSAS. The presence of low vitamin D levels may fail to modulate the inflammatory response in OSAS patients, resulting in the overexpression of inflammatory markers.

Future, more extensive prospective studies are needed to confirm such preliminary findings and shed further light on this complex issue.

## Figures and Tables

**Table 1:** Variables analyzed in the OSAS patients included in the study.

OSAS Cases, *n*	127
Males, *n* (%)	77/127 (60.6)
Median (IQR) age, years (*n* = 127)	5 (4–7)
Median (IQR) weight, kg (*n* = 127)	18.9 (15.3–26.0)
Median (IQR) height, cm (*n* = 127)	112 (102–123)
Median (IQR) BMI, kg/m^2^ (*n* = 127)	15.4 (14.4–17.4)
Median (IQR) BMI percentile (*n* = 127)	30.5 (12–72.5)
Mean (SD) 25(OH)D, ng/mL	22.4 (7.7)
Median (IQR) IgE total, UI/mL (*n* = 118)	38.5 (10–168)
Median (IQR) MPV, fL (*n* = 127)	6.9 (6.6–7.4)
Median (IQR) PLT ×10^3^/mcL (*n* = 127)	320 (258–383)
Median (IQR) AHI, events/h (*n* = 127)	7.2 (5.1–12.1)
Tonsil size grading III–IV, *n* (%)	78/127 (61.4)
Friedman palate position III–IV, *n* (%)	51 (40.2)
Median (IQR) oxygen saturation, % (SpO_2_ nadir) (*n* = 127)	92 (89–93)
Oral breathing, *n* (%)	78/127 (61.4)
Nasal airway patency, *n* (%)	104/127 (81.9)
Snoring, *n* (%)	112/127 (88.2)
Allergen Sensitization, *n* (%)	32/127 (25.2)
Asthma, *n* (%)	22/127 (17.3)
Rhinitis, *n* (%)	53/127 (41.7)

BMI: Body Mass Index. 25(OH)D: serum 25-hydroxyvitamin D. PLT: Platelet count. MPV: Mean Platelet Volume. AHI: Apnea–Hypopnea Index.

**Table 2:** Demographic and auxological data and serum 25(OH)D levels for OSAS patients and healthy controls (HC) matched by age.

	HC (*n* = 96)	OSAS (*n* = 96)	*p*-Value
Males, *n* (%)	48 (50.0)	60 (62.5)	0.08
Median (IQR) age, years (*n* = 192)	6 (4–8)	6 (4–8)	0.86
Median (IQR) weight, kg (*n*= 192)	18.9 (15.7–25.5)	20 (16–28.4)	0.20
Median (IQR) height, cm (*n* = 192)	112.8 (102.5–127.0)	116 (105–127)	0.34
Median (IQR) BMI, kg/m^2^ (*n* = 192)	15.5 (14.3–16.8)	15.7 (14.4–18)	0.19
Median (IQR) BMI percentile (*n* = 192)	29.5 (9.5–61.5)	29.1 (10.5–74.0)	0.33
Mean (SD) 25(OH)D, ng/mL	25.5 (8.7)	22.4 (7.6)	0.009

BMI: Body Mass Index. 25(OH)D: serum 25-hydroxyvitamin D.

**Table 3:** Body Mass Index percentile, platelet count, Mean Platelet Volume, Apnea–Hypopnea Index, tonsil size grading, and SpO_2_ nadir in OSAS patients with serum 25(OH)D levels <20 ng/mL and in OSAS patients with levels ≥20 ng/mL.

	25(OH)D ≥ 20 ng/mL (*n* = 83)	25(OH)D < 20 ng/mL (*n* = 44)	*p*-Value
Median (IQR) BMI percentile	38 (12–74)	23.3 (9.8–59.8)	0.22
Median (IQR) PLT ×10^3^/mcL	297 (246–371)	339.5 (296–411.5)	0.008
Median (IQR) MPV, fL	6.9 (6.6–7.4)	6.9 (6.4–7.4)	0.35
Median (IQR) AHI, events/h	6.5 (5.1–10.8)	9.3 (5.4–13.2)	0.10
Tonsil size grading III–IV, *n* (%)	47 (56.6)	31 (70.5)	0.12
Median (IQR) SpO_2_ nadir, %	92 (90–94)	91 (86.5–92.5)	0.08

BMI: Body Mass Index. PLT: platelet count. MPV: Mean Platelet Volume. AHI: Apnea–Hypopnea Index.

**Table 4:** Correlations between serum 25(OH)D levels and Body Mass Index percentile, platelet count, Mean Platelet Volume, Apnea–Hypopnea Index, SpO_2_ nadir in OSAS patients.

	25(OH)D, ng/mL
	rho (*p*-Value)
BMI percentile	0.05 (0.58)
PLT ×10^3^/mcL	−0.25 (0.005)
MPV, fL	0.06 (0.49)
AHI, events/h	−0.14 (0.11)
SpO_2_ nadir, %	0.14 (0.11)

BMI: Body Mass Index. PLT: platelet count. MPV: Mean Platelet Volume. AHI: Apnea–Hypopnea Index.

**Table 5:** Apnea–Hypopnea Index and serum 25(OH)D levels in OSAS patients with and without allergen sensitization.

		Allergen Sensitization		*p*-Value
		NO (*n* = 95)	YES (*n* = 32)	
AHI, *n* (%)	AHI < 5 events/h	19 (20.0)	8 (25.0)	0.83
	5≤ AHI < 10 events/h	42 (44.2)	13 (40.6)	
	AHI ≥ 10 events/h	34 (35.8)	11 (34.4)	
Median (IQR) AHI, events/h		7.3 (5.3–12.8)	6.9 (4.9–10.9)	0.56
25(OH)D < 20 ng/mL, *n* (%)		30 (31.6)	14 (43.8)	0.21
Mean (SD) 25(OH)D, ng/mL		22.7 (7.9)	21.4 (7.3)	0.43

AHI: Apnea–Hypopnea Index.

## Data Availability

The datasets generated and/or analyzed during the current study are available from the corresponding author upon reasonable request.

## Abbreviations

OSAS	obstructive sleep apnea syndrome
AHI	apnea–hypopnea index
ODI	oxygen desaturation index
PG	polygraphy
HRP	home cardio-respiratory polygraphy
25(OH)D	serum 25-hydroxyvitamin D
MPV	mean platelet volume
PLT	platelet count
BMI	body mass index
IQR	interquartile range
